# Role of PTCH1 gene methylation in gastric carcinogenesis

**DOI:** 10.3892/ol.2014.2178

**Published:** 2014-05-26

**Authors:** YUN ZUO, YU SONG, MIN ZHANG, ZHEN XU, XIAOLAN QIAN

**Affiliations:** Department of Oncology, The First Hospital of Zhangjiagang, Zhangjiagang, Jiangsu 215600, P.R. China

**Keywords:** gastric cancers, PTCH1 gene, hedgehog signal pathway, methylation, 5-aza-2′-deoxycytidine

## Abstract

The present study aimed to investigate the role of PTCH1 methylation in gastric carcinogenesis and the therapeutic effect of the methylation inhibitor, 5-aza-2′-deoxycytidine (5-aza-dC), in the treatment of gastric cancer. Total RNA was extracted from 20 gastric cancer tissues, their corresponding adjacent normal tissues and a gastric cancer AGS cell line. PTCH1 mRNA expression was detected by quantitative PCR, and the PTCH1 methylation of the promoter was examined by methylation-specific PCR. The AGS cells were treated with 5-Aza-dC; apoptosis and the cell cycle were examined by flow cytometry, and the PTCH1 methylation level was observed. PTCH1 expression was negatively correlated with promoter methylation in the gastric cancer tissues, their corresponding adjacent normal tissues and the gastric cancer AGS cell line (r=−0.591, P=0.006). 5-Aza-dC treatment caused apoptosis and the G_0_/G_1_ phase arrest of the AGS cells, and also induced the demethylation and increased expression of PTCH1. In conclusion, the study found that the hypermethylation of the PTCH1 gene promoter region is one of the main causes of low PTCH1 expression in AGS cells. Demethylation agent 5-Aza-dC can reverse the methylation status of PTCH1 and regulate the expression of PTCH1, indicating its potential role in gastric cancer treatment.

## Introduction

The hedgehog (Hh) signaling pathway is one of the most significant molecular mechanisms for the regulation of the embryonic developmental process, and its activation shows an association with the emergence of a number of solid tumors (1,2). Studying the function and regulatory mechanism of the HH signaling pathway may be further elucidate the mechanisms underlying the development of malignant tumors and aid in their diagnosis and treatment. The Hh family mainly includes SHH, HHIP, PTCH1, Smo and Gli, where PTCH1 is a negative regulatory factor of the Hh signaling pathway. The Hh pathway could be activated by inhibiting the expression of PTCH1 and could also be involved in tumorigenesis. It has been reported that the PTCH1 gene could be methylated and involved in the formation of certain tumors (3). However, the association between PTCH1 methylation and gastric cancer is rarely reported. The purpose of the present study was to investigate the role of PTCH1 hypermethylation on gastric carcinogenesis by observing PTCH1 gene methylation and expression in gastric cancer tissues and the gastric cancer AGS cell line, as well as by investigating the effect of the demethylating agent, 5-aza-2′-deoxycytidine (5-Aza-dC), on PTCH1 gene methylation and expression in gastric cancer cells.

## Materials and methods

### Specimens and cell culture

A total of 20 gastric cancer tissues and their corresponding adjacent normal tissues were collected from 20 gastric patients who underwent curative resections. These cancer tissue specimens and adjacent normal tissue specimens were routinely confirmed by biopsy and stored in liquid nitrogen. The study group consisted of 14 male and 6 female patients, with a median age of 60.12 years. No primary tumors from other sites were observed for these gastric patients. Prior to surgery, the gastric patients received no other treatment. The human gastric cancer AGS cell line was purchased from the cell center of Shanghai Life Science Institutes of the Chinese Academy of Sciences (Shanghai, China) and was cultured with 10% fetal bovine serum (Hyclone, Shanghai, China) and Ham’s F12K medium Sigma-Aldrich (St. Louis, MO, USA) at 37°C and 5% CO_2_. This study was performed at the First Hospital of Zhangjiagang (Zhangjiagang, China) and also approved by the Institutional Review Board of the First Hospital of Zhangjiagang. Written informed consent was obtained from each patient.

### Instruments and reagents

5-Aza-2′-deoxycytidine (5-Aza-dc) was purchased from Sigma-Aldrich, and TRIzol reagent was bought from Invitrogen Life Technologies (Carlsbad, CA, USA). The RNA reverse transcription kit, propidium iodide (PI) and Annexin-V/PI double-staining streaming apoptosis detection kits were purchased from Shanghai Jingmei Biological Engineering Co., Ltd. (Shanghai, China). The methylation conversion kit, EZ DNA Methylation-Gold™, was purchased from the Beijing Science and Technology Development Co., Ltd. (Beijing, China). The ABI7500 Real-Time PCR instrument was manufactured by Applied Biosystems (Life Technologies, Carlsbad, CA, USA).

### AGS cell treatment with 5-Aza-dC

AGS cells (3×10^5^) were plated in 100-ml flasks with Ham’s F12K medium containing 10% fetal bovine serum at 37°C with 5% CO_2_. Next, 24 h after the cells had reached the logarithmic growth phase, they were treated with 5×10^−6^ mol/l 5-Aza-dc. The treatment medium was changed every 24 h for 3 times, then the cells were collected. Control cell groups without 5-Aza-dc treatment were also collected for comparison.

### Total RNA extraction, cDNA synthesis and DNA extraction

Total RNA (dissolved with 50 μl Tris-EDTA) was extracted by a conventional method once 100 mg tissue in liquid nitrogen had been ground into a powder, or after 1×10^6^ AGS cells had been washed with phosphate-buffered saline (PBS) and collected by centrifugation at 12,000 rpm for 15 min. The concentration and purity of the RNA were tested by electrophoresis. To synthesize cDNA, 1 μl 0.5 μg/μl Oligo-(dT)_18_ and 10 μl DEPC-H_2_O were added to 1 μl total RNA and mixed, and then bathed in 70°C water for 5 min and cooled rapidly on ice. The following reagents were then added: 4 μl 5× reaction buffer, 1 μl 20 U/μl RNA enzyme inhibitors and 2 μl 10 mmol/l dNTP. The mixture was held in a water bath at 37°C for 5 min, then 1 μl 200 U/μl reverse transcriptase (M-MuLV) was added and the mixture was placed in a 42°C water bath for 60 min. The process was completed with immersion in a 70°C water bath for 10 min. The synthesis reaction was terminated. Synthesized cDNA product were stored at −20°C. The DNA of the tumor tissues and cells were obtained using the phenol/chloroform extraction method. DNA quality was measured with a NanoDrop-1000 full wavelength UV/VIS scanning spectrophotometer (Thermo Fisher Scientific, Waltham, MA, USA).

### PTCH1 gene quantitative (q)PCR analysis

PTCH1 gene qPCR analysis was started with 2 μl cDNA product as a template, adding 0.5 μl for each PTCH1 upstream and downstream primer (Shanghai Sangon Biological Engineering Technology and Services Co, Ltd., Shanghai, China; Table I), as well as 10 μl 2× SYBR Green Real Time PCR Master Mix liquid (Shanghai GeneCore BioTechnologies Co., Ltd., Shanghai, China) and 7 μl sterile water. Next, detection of 35 amplification loops were conducted with the ABI7500 Real-Time PCR instrument at conditions of 95°C for 5 sec, 55°C for 5 sec and 72°C for 30 sec. The melting curves of the amplified products were analyzed, using β-actin as an internal reference. The relative expression of the PTCH1 gene was calculated by 2^−ΔΔCt^ and analyzed by agarose gel electrophoresis.

### Cell cycle and apoptosis detection by flow cytometry

Treated and untreated AGS cells (~1×10^6^ cells each) were collected and centrifuged at 1000 rpm for 5 min, and then the culture medium was discarded. The cells were washed once with 3 ml 0.01 mol/l PBS (pH 7.4), which was then removed by centrifugation at 1,500 rpm for 10 min. The cells were fixed for 24 h at 4°C by adding 1 ml ice-cold 70% ethanol. The fixative was centrifuged at 1,500 rpm for 10 min and discarded again. The cells were resuspended with 3 ml PBS for 5 min. The cells were filtered once with a 400-mesh screen and the PBS was removed by centrifugation. The cells were stained with 1 ml PI dye (final concentration at 1,500 rpm for 10 min of 100 μg/ml, 0.01 mol/l PBS, pH 7.4) and stored at 4°C in the dark for 30 min. A flow cytometer was used to detect the cell cycle and apoptosis (BD FACSCalibur, BD Biosciences, Franklin Lakes, NJ, USA).

### DNA bisulfite conversion

DNA bisulfite conversion was conducted using the EZ DNA Methylation-Gold kit, according to the manufacturer’s instructions. Briefly, 130 μl CT Conversion Reagent was added to 20 μl DNA (500 ng), then mixed and maintained at 98°C for 10 min. The sample was then held at 64°C for 2.5 h. Storage was at 4°C. A total of 600 μl M-Binding Buffer was added into an activated Zymo-spin column. The converted DNA that was stored at 4°C was then added into the spin column and the content was mixed by inversion. Centrifugation at full-speed (12,000 rpm) was applied for 30 sec, then the effluent was removed and 100 μl M-Wash Buffer was added into the column. Centrifugation at 12,000 rpm was applied for 30 sec and then 200 μl M-Desulphonation Buffer was added into the column prior to storage for 15–20 min and centrifugation again at full speed (12,000 rpm) for 30 sec. A total of 200 μl M-Wash Buffer was added into the column and centrifugation at 12,000 rpm was applied for 30 sec. Another 30 μl M-Elution Buffer was added, and then the transformed DNA was collected by centrifugation at 12,000 rpm and stored at −20°C for 1 week.

### Methylation-specific PCR (MSP) detection

CpG island analysis and primer design for PTCH1 mRNA of the transcription start site (counted as 0:00) −3950 bp upstream and +2050 bp downstream were conducted with Methyl Primer Express^®^ v1.0 software (Applied Biosystems; Life Technologies). Table I lists the MSP primers. PCR cooling amplification was performed in 8 μl bisulfite-treated DNA with a conventionally configured reaction system. Briefly, pre-degeneration was completed at 95°C for 5 min. Prior to PCR, predegeneration was completed at 95°C for 5 min. Next, cooling PCR was performed for 10 cycles (PCR degeneration at 94°C for 30 sec, with renaturation temperature decreases of 0.5°C per cycle; temperature decreases from +3°C to −2°C for 30 sec; and extension at 72°C for 30 sec). Next, the normal PCR was performed for 40 cycles (PCR degeneration at 94°C for 30 sec; −2°C renaturation for 30 sec; and extension at 72°C for 30 sec). Amplification products were analyzed by 1.5% agarose gel electrophoresis.

### Statistical analysis

Data were analyzed with SPSS 13.0 statistical software (SPSS, Inc., Chicago, IL, USA). A non-parametric Mann-Whitney U test was applied to compare the differences in relative PTCH1 mRNA expression between the gastric carcinoma and adjacent normal tissues. Fisher’s exact test was applied to compare the differences in PTCH1 gene promoter methylation rate between the gastric cancer and adjacent normal tissues. Spearman’s test was used to study the correlation between PTCH1 methylation and its expression in gastric cancer tissues.

## Results

### Expression of PTCH1 mRNA in the gastric cancer AGS cell line and gastric cancer and adjacent normal tissues

The expression of the PTCH1 gene in the gastric cancer tissues and gastric cancer AGS cell line were observed by qPCR. Taking AGS as a reference sample, the relative expression levels of PTCH1 in the gastric cancer and adjacent normal tissues were 1.26±0.89 and 2.74±1.67, respectively. There was a significant statistical difference between the two groups (n=20; P=0.023).

### Methylation changes in the PTCH1 gene promoter region of gastric cancer and adjacent normal tissues, detected using MSP

**Figure 1:**
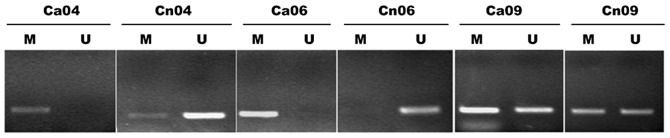
Products of methylation-specific PCR (MSP) analysis of PTCH1 gene promoter methylation in gastric cancer and adjacent normal tissues. Ca, gastric cancer; Cn, adjacent normal tissue; M, methylated PCR products; U, unmthylated PCR products.

In order to observe the incidence of PTCH1 gene methylation in the gastric cancer tissues, MSP detection was conducted in the gastric cancer and adjacent normal tissues of 20 patients. Methylation amplified bands were observed in 12 cancer tissues and 4 adjacent normal tissues, with an incident rate of 60% and 20%, respectively (Fisher’s exact probability test; two-tailed test P=0.057 and one-tailed test P=0.029). This indicated the presence of high PTCH1 gene methylation in the gastric cancer tissues. Fig. 1 shows typical electrophoretograms.

### Correlation between PTCH1 methylation and expression in gastric cancer tissues

In order to further study the association between PTCH1 expression and promoter methylation, the correlation between PTCH1 gene methylation and its relative expression in gastric cancer and adjacent normal tissues was analyzed in 20 gastric cancer patients. The qPCR values of PTCH1 in methylated and unmethylated tissues were 0.93±0.71 and 2.58±1.52, respectively. The expression of PTCH1 was negatively correlated with the methylation status, with a correlation coefficient of −0.591 (P=0.006).

### Impact of 5-Aza-dC treatment on the cell cycle, the apoptosis of AGS cells and the PTCH1 gene methylation status

**Figure 2:**
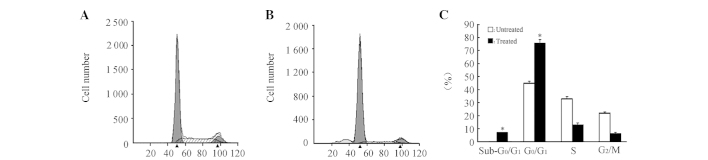
Cell cycle changes in gastric cancer AGS cells treated with 5-Aza-dC, as detected by flow cytometry. (A) Untreated cells. (B) Treated cells. (C) The percentage of cells within the different phases of the cell cycle (^*^P<0.05 vs. untreated cells; n=3, mean ± standard deviation). 5-Aza-dC, 5-aza-2′-deoxycytidine.

**Figure 3:**
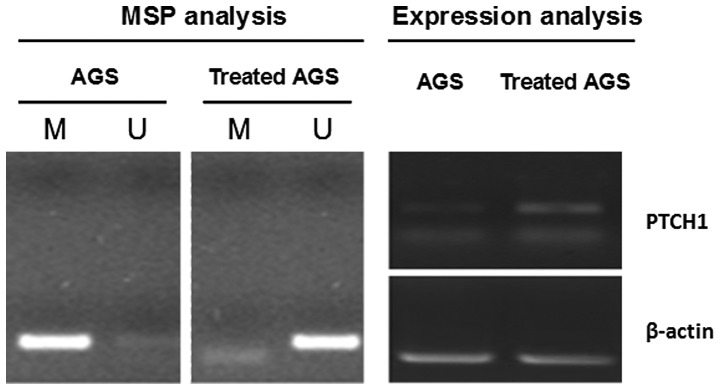
PTCH1 methylation and PTCH1 mRNA expression of gastric cancer AGS cells treated with 5-Aza-dC. M, methylated PCR products; U, unmethylated PCR products; MSP, methylation-specific PCR; 5-Aza-dC, 5-aza-2′-deoxycytidine.

PI staining was conducted in the AGS cells at the logarithmic phase at 72 h post-treatment with 5×10^−6^ mol/l 5-Aza-dC, then the cell cycle of the treated AGS cells was analyzed (Fig. 2). A G_0_/G_1_ block was observed in the treated AGS cells. Meanwhile, Annexin V/PI double-staining was conducted to detect the cell apoptosis of the treated AGS cells. Significant levels of apoptosis were observed in the treated AGS cells. There were significant differences between the treated group and the control group according to the results of three independent experiments (P<0.05; Fig. 2). MSP and relative expression measurements were conducted on PTCH1 gene methylation and expression in the AGS cells prior to and following the treatment with 5-Aza-dC AGS, as shown in Fig. 3 (4). Amplification was obtained for the methylated sequence of the PTCH1 gene, but not for the demethylated sequence in the AGS cells prior to the treatment. The relative PTCH1 mRNA expression was low in the AGS cells prior to the treatment. Amplification was obtained for the demethylated sequence of the PTCH1 gene, but not for the methylated sequence in the AGS cells following the treatment. The relative expression of the PTCH1 mRNA was increased in the AGS cells following the treatment. This further indicates the negative correlation between PTCH1 gene hypermethylation and expression.

## Discussion

Cancer epigenetics studies have found that there is widespread hypomethylation and partial regional hypermethylation of CpG islands in the genomic DNA of cancer cells. Partial regional hypermethylation of CpG islands may result in the inactivation of certain tumor suppressor genes, which is an important mechanism for causing the malignant transformation of cells (5,6). Demethylation agents, such as 5-Aza-dC, could be used to obtain the re-expression of these hypermethylation genes and to play a significant role in tumor suppression (7).

The Hh signaling pathway is a crucial signal transduction pathway in the regulation of embryonic development. The Hh signal is most active in the embryonic formation period, and has no expression or extremely low expression in normal mature tissues. However, aberrant activation of the Hh signal transduction pathway in cells of mature tissues and organs can result in various diseases and tumorigenesis. The high expression of SHH in small cell lung cancer tissues and cell lines has been reported in a previous study (8).

In total, 81% of tumor cell lines from the digestive tract (including the esophagus, stomach, bile duct and pancreas) have been shown to express SHH and its receptor, PTCH1 (9). The hypomethylation of HHIP, the inhibitor of SHH, has been observed in gastrointestinal tumors (10). The missing or mutated PTCH1 gene has rarely been reported in gastric cancer in previous studies. Few studies have also reported the correlation between the methylation of the PTCH1 gene and gastric cancer. In the present study, the expression of PTCH1 mRNA was detected in gastric cancer tissues, adjacent normal tissues and a human gastric cancer cell line, with a relatively high expression of PTCH1 mRNA in the adjacent normal tissues compared with the cancer tissues. High methylation levels of PTCH1 were observed in the gastric cancer tissues and the cancer cell lines. This indicates that the high methylation modification of PTCH1 as a tumor suppressor gene may be one of the main mechanisms of gastric cancer.

In conclusion, the hypermethylation of the PTCH1 gene promoter region in gastric cancer was observed in the present study. Following this preliminary result, further studies of molecular mechanisms involved in regulating the PTCHI methylation changes and the association between PTCH1 hypermethylation and the biological features of gastric cancer are required. More scientific experimental evidence regarding PTCH1 gene hypermethylation as a gastric cancer marker and its function in guiding the treatment and prognosis of gastric cancer are also required from these studies.

## Figures and Tables

**Table I:** Primer sequences and length of PCR product.

PCR type	Primer name	Primer sequences	Product length, bp
qPCR	PTCH1	5′-TGTGCGCTGTCTTCCTTCTG-3′ 5′-ACGGCACTGAGCTTGATTC-3′	119
	β-actin	5′-GCCATCCTGCGTCG-3′ 5′-TGGGCACCGGAACCGCT-3′	260
MSP	Methylation	5′-GTTAATTCGTGATTTTTCGGA-3′ 5′-ATAACAAACCTACGAACCGC-3′	197
	Unmethylation	5′-AATGTTAATTTGTGATTTTTTGGA-3′ 5′-TAAATAACAAACCTACAAACCAC-3′	197

qPCR, quantitative PCR; MSP, methylation-specific PCR.
